# Supplementary material for: Identification of photoperiod-induced specific miRNAs in the adrenal glands of Sunite sheep (Ovis aries)
Source: Front Vet Sci. 2022 Jul 22;9:888207. doi: 10.3389/fvets.2022.888207 (PMC9354845; doi:10.3389/fvets.2022.888207)
Supplement: Supplementary file 1 [file Data_Sheet_1.ZIP › Supplementary file/miRDeep2 file.zip!/miRDeep2 file/Novel_Structure/SP42A3/Novel_427.pdf]

Provisional ID	:	NC_019462.2_11636
Score total	:	0.1
Score for star read(s)	:	-1.3
Score for read counts	:	-0.8
Score for mfe	:	0.7
Score for randfold	:	1.6
Score for cons. seed	:	
Total read count	:	10
Mature read count	:	6
Loop read count	:	0
Star read count	:	4

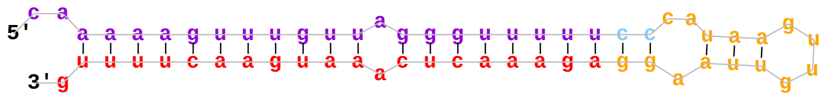

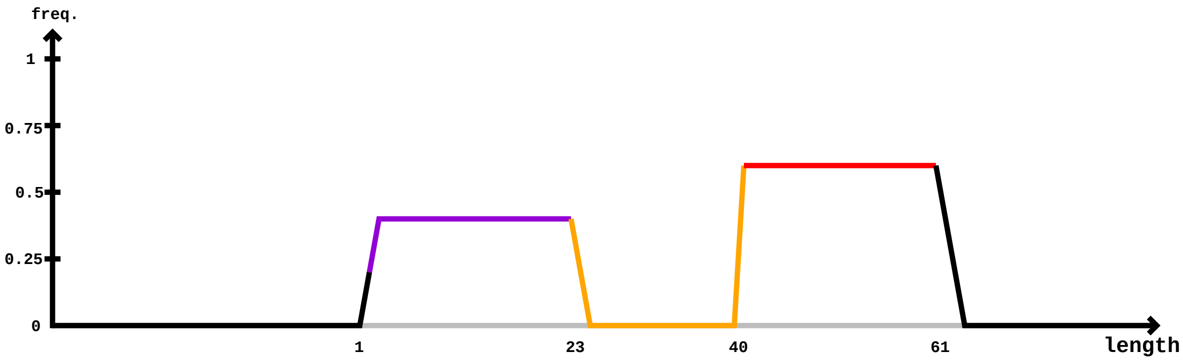

Star Mature

```
5'- ccuuuuguaauguuuuuucuacuggauuagccaaaaaguuuguuagggguuuuucccauaaguuguuaaggagaaacucaaaugaacuuuuggaccaacccaguauauuccagu   -3'   obs
    ccuuuuguaauguuuuuucuacuggauuagccaaaaaguuuguuagggguuuuucccauaaguuguuaaggagaaacucaaaugaacuuuuggaccaacccaguauauuccagu         exp
    ..................((((((.((.(((((((.(((((((.((((((((((..(((....))).)))))))))).))))))))))))).).)).))))))........   reads   mm   sample
    ...............................caaaaaguuuguuUggguuuuu............................................................   2       1    seq
    ................................aaaaaguuuguuCggguuuuu............................................................   1       1    seq
    ................................aaaaaguuuguuGggguuuuuc...........................................................   1       1    seq
    ......................................................................agaaacucaaaugaacuuuug......................   3       0    seq
    ......................................................................agaaacucaaaugaacuuuugg.....................   1       0    seq
    ......................................................................agaaacucaaaugaacuuuugga....................   2       0    seq
```
